# The Role of Mechanical Economy and Efficiency as Key Drivers of Cardiac Rehabilitation Exercise Performance Improvement in a Patient With Heart Failure and an Implanted CRT‐D: A Case Report and Literature Review

**DOI:** 10.1002/ccr3.71315

**Published:** 2025-10-16

**Authors:** Javier Loureiro Diaz, Praveen Jayaprabha Surendran, Prasobh Jacob, Salma Chbib, Amine Ghram, Liam David Foster, Omar Ibrahim

**Affiliations:** ^1^ Performance and Health Group, Department of Physical Education and Sport, Faculty of Sports Sciences and Physical Education Universidade da Coruña Oleiros Spain; ^2^ Cardiac Rehabilitation Department Heart Hospital. Hamad Medical Corporation Doha Qatar; ^3^ Pharmacy Department Heart Hospital. Hamad Medical Corporation Doha Qatar; ^4^ Research Laboratory “Heart Failure, LR12SP09” Hospital Farhat HACHED of Sousse Sousse Tunisia; ^5^ Healthy Living for Pandemic Event Protection (Hl‐Pivot) Network Chicago Illinois USA; ^6^ Rehabilitation Department The View Hospital Doha Qatar

**Keywords:** cardiac rehabilitation, cardiac resynchronization therapy device, cardiopulmonary exercise testing, cardiovascular diseases, exercise capacity, heart failure, mechanical economy, mechanical efficiency

## Abstract

Heart failure with reduced ejection fraction leads to exercise intolerance due to central and peripheral dysfunction. Improvements in exercise performance are not fully captured by traditional measures like peak oxygen consumption alone. A 57‐year‐old male with a complex cardiac medical history of hypertrophic cardiomyopathy with left ventricle non‐compaction, Wolff‐Parkinson‐White syndrome, transient ischemic stroke, and heart failure with reduced ejection fraction with an implanted cardiac resynchronization therapy defibrillator underwent cardiac rehabilitation. Cardiopulmonary exercise testing revealed a non‐proportional improvement in peak oxygen consumption (0.3 METs, 9%) versus peak workload (48 W, 61%). Gross mechanical economy and efficiency improved at all physiological thresholds: at ventilatory threshold by 11%, at respiratory compensation point by 60% and 41%, and at peak oxygen consumption by 47% and 33%, respectively. Mechanical economy and mechanical efficiency should be measured and reported for assessing rehabilitation outcomes in heart failure with reduced ejection fraction.

## Graphical abstract

Conceptual framework linking Mechanical Economy (MEC) and Mechanical Efficiency (MEF) to clinical phenotypes and training response in a 57‐year‐old male patient with HFrEF and a CRT‐D. After exercise‐based cardiac rehabilitation peak workload increased disproportionately to modest changes in peak oxygen consuption. MEC/MEF improved at physiological thresholds and peak effort. MEC and MEF reveal disproportionate gains in external work for metabolic cost and should be reported alongside traditional cardiopulmonary outcomes in cardiac rehabilitation.

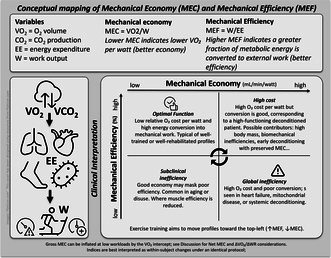

**Summary**

- Mechanical economy and mechanical efficiency offer a plausible hypothesis to the discrepancy between peak workload change and the change in peak oxygen consumption pre‐to‐post cardiac rehabilitation in heart failure patients.
- Both assess indirectly physiological changes from exercise interventions that may not be captured by traditional parameters such as peak oxygen consumption alone.

## 1 Introduction

Heart failure with reduced ejection fraction (HFrEF) is characterized by a reduced ability of the heart to pump blood effectively, leading to exercise intolerance [1]. Peripheral changes due to poor perfusion among other factors result in a reduction in skeletal muscle mass and contractile function, and an increased oxygen cost of force generation, leading to early fatigue and exercise intolerance [1, 2, 3]. Overall, HFrEF results in decreased quality of life, increased hospital readmissions, and increased morbidity and mortality [1, 2, 4, 5].

Exercise is a core component of cardiac rehabilitation (CR) and is a well‐established therapy for patients with HFrEF (Class 1‐A recommendation) [6, 7]. CR lowers the risk of readmission and mortality, and can improve symptoms and quality of life [2, 5]. These benefits are greatly attributed to the improvement in exercise capacity [5, 8].

Cardiac resynchronization therapy defibrillator (CRT‐D) is a cardiac implantable electronic device used in the treatment of cardiac arrhythmias. CRT‐D improves symptoms and health‐related quality of life [8], reduces the incidence of sudden cardiac death [9, 10], and lowers the risk of mortality and hospital readmission [11, 12, 13, 14] in patients with heart failure. Patients with CRT‐D engaged in CR may also experience improvements in exercise capacity, left ventricular dimensions, and ejection fraction measured by echocardiography [15].

Cardiopulmonary exercise testing (CPET) is the gold standard of measuring peak oxygen consumption (*V*O_2peak_) and assessing exercise performance [16]. It provides in‐depth information about the cardiopulmonary responses to exercise, the mechanisms of exercise limitation, and the effects of exercise training in patients with HFrEF [17, 18].

**FIGURE 1:**
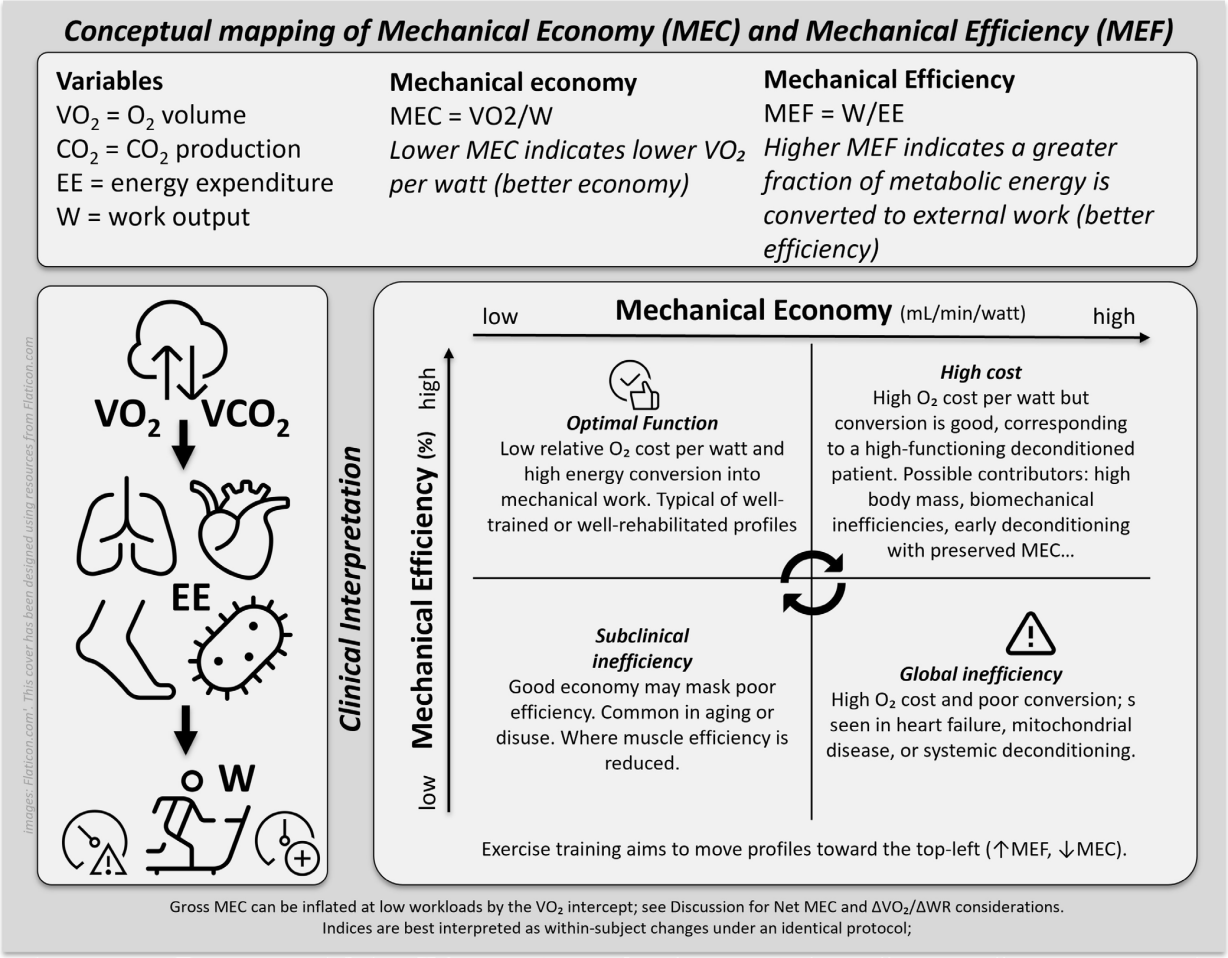
Conceptual mapping of mechanical economy (MEC) and mechanical efficiency (MEF) with clinical phenotypes and their clinical interpretation. EE, energy expenditure; MEC, mechanical economy (*V*O_2_/W; mL·min^−1^·W^−1^); MEF, mechanical efficiency (gross, W/EE × 100, %); VCO₂, carbon dioxide output; *V*O_2_, oxygen uptake; W, external work (Watts); Δ*V*O_2_/ΔWR, slope of oxygen uptake versus work‐rate (mL·min^−1^·W^−1^).

Mechanical economy (MEC) and mechanical efficiency (MEF) are both measures of how efficiently the human body converts metabolic energy into mechanical work (W) [19]. They are related but distinct concepts in exercise physiology (Figure 1). MEC is essentially the ratio of energy input needed during exercise (i.e., oxygen consumption) to produce external W. MEC can indirectly be inferred by the relationship *V*O_2_/W [20]. MEF is a measure of how efficiently the body can produce W for a given amount of energy expenditure (EE). EE can be derived from gas analysis during CPET [21, 22]. MEF (W/EE) is considered a measure of how effectively an individual converts EE into W [23]. A lower MEF may indicate a higher energy cost during exercise or that more energy is inefficiently used or wasted.

MEC and MEF are well‐described in the context of exercise physiology and sport performance; however, their integration into CR reports is limited. We applied both variables as primary outcome indicators after individualized rehabilitation to highlight their clinical utility in characterizing submaximal improvements and cardiorespiratory‐muscular adaptations. HFrEF‐related physiological changes can lead to a worsening of both MEC and MEF, resulting in early fatigue and exercise intolerance. Improving MEC and MEF can reduce the metabolic cost of exercise, allowing patients to perform more work with less oxygen cost and/or less EE [1, 20, 24]. This can lead to enhanced functional capacity, reduced symptoms, and improved quality of life [5, 25, 26]. The effects of CR exercise training on MEC and MEF in coronary artery disease patients have been studied [24], but MEC and MEF are underexplored in HFrEF.

This case report highlights MEC and MEF for the detection of physiological dysfunction and the assessment of physiological adaptations in response to training in a HFrEF patient with a CRT‐D in DDD mode during treadmill exercise. CARE guidelines were used to report this case.

## 2 Case History/Examination

A 57‐year‐old male with a history of HFrEF (left ventricular ejection fraction 28%), hypertension, smoker with an 18 pack‐year history, chronic kidney disease, and a prior transient ischemic stroke was referred for outpatient CR. His cardiac history included hypertrophic cardiomyopathy with left ventricle non‐compaction, Wolff‐Parkinson‐White syndrome treated with ablation, and an implantable cardioverter defibrillator, later upgraded to CRT‐D in DDDR rate responsive mode. The patient presented in New York Heart Association functional class II and reported total avoidance of physical activity due to concerns about exercise safety. His medical treatment included Amiodarone 200 mg, once daily; Bisoprolol 3.75 mg, once daily; Sacubitril/Valsartan 100 mg, bis in die; Furosemide 80 and 40 mg, bis in die; Spironolactone 25 mg, once daily; Atorvastatin 20 mg, once daily; Warfarin adjusted based on INR readings; Ferrous Fumarate 200 mg, once daily; Ergocalciferol 50,000 units, once weekly; and Pantoprazole 40 mg, once daily.

## 3 Differential Diagnosis

**FIGURE 2:**
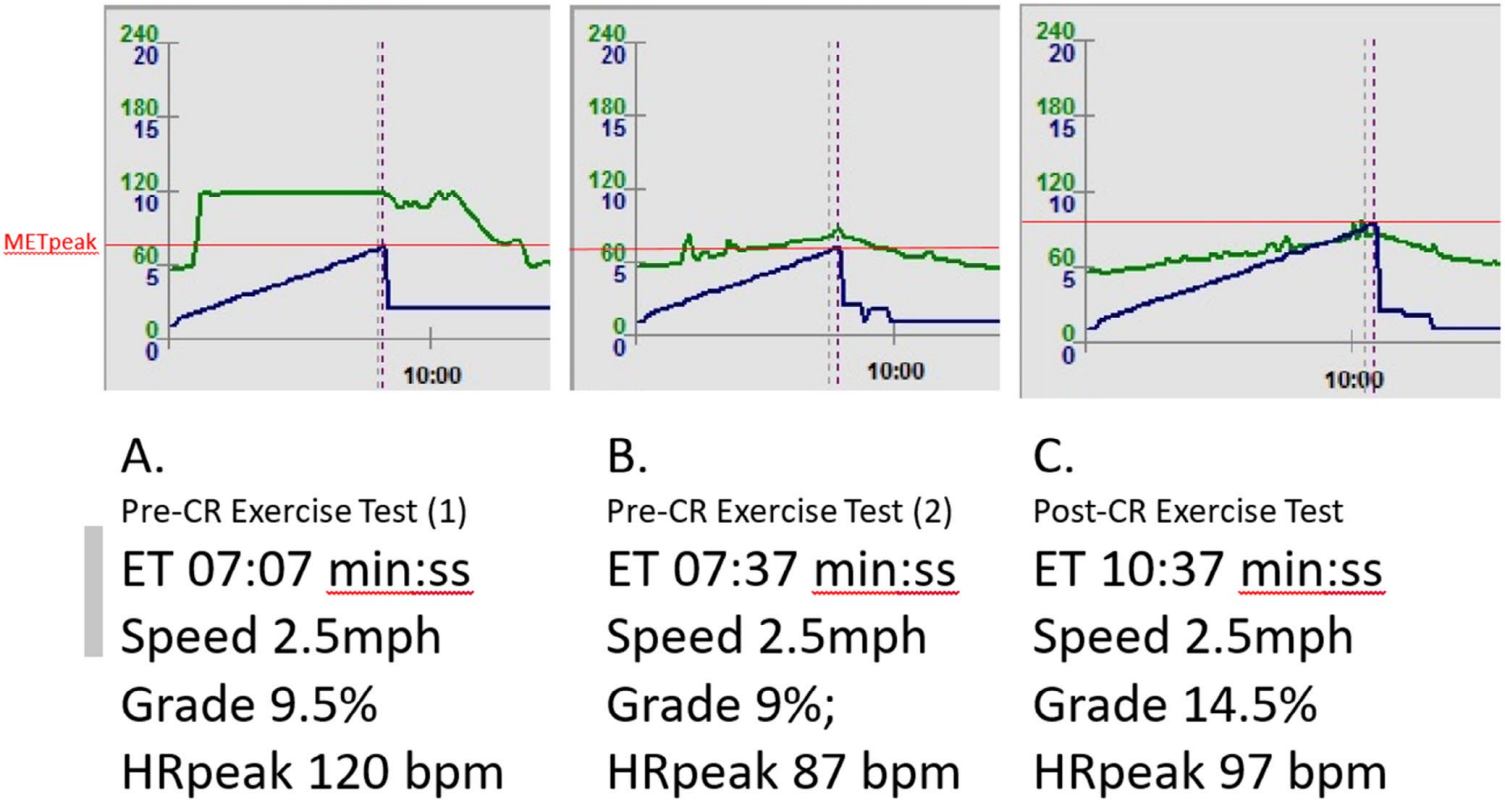
Heart rate and increasing workload during exercise testing with CRT‐D on DDDR versus DDD mode. bpm, beats per minute; CR, cardiac rehabilitation; ET, exercise time; HRpeak, peak heart rate; MET, metabolic equivalent of task; min:ss; minutes and seconds; MPH, miles per hour.

Treadmill exercise testing is the preferred testing modality in patients with rate response devices [27]. Initially, a 12‐lead electrocardiographically monitored treadmill ramp protocol was performed to assess heart rate response to exercise with CRT‐D in DDDR mode. CRT‐D was working too aggressively for the patient, with minimal change in heart rate response (Image A in Figure 2). The patient also reported exercise‐related fatigue and breathlessness out of proportion to the workload, despite optimal medical therapy.

This device's response can be mostly attributed to chronotropic incompetence and inappropriate sensor function for rate‐adaptive pacing (under or over responsive) [27]. If ventricular pacing is needed or desired, the device should be programmed to at least track the maximum sinus rate reached during exercise. However, there is still much controversy about the effectiveness of rate‐responsive devices, especially in patients diagnosed with heart failure. As no deleterious effects have been described, the rate response feature can probably be programmed on or off safely. Device interrogation showed minimal heart rate fluctuations during daily activity suggesting the rate‐responsive algorithm may have been insufficiently adapting to physiological needs or even contributing to dysynchrony. Following multidisciplinary discussion with the electrophysiology team, the CRT‐D was reprogrammed to DDD mode to allow for the patient's own intrinsic increase in heart rate to meet the exercise demand. The aim was to optimize ventricular synchrony by maintaining a consistent, optimal delay between sensed or paced atrial and ventricular events, minimizing beats that disrupt effective resynchronization and cardiac output under controlled pacing conditions. This approach is especially relevant in heart failure patients where electromechanical synchrony is critical for both ventricles to be reliably paced together for functional recovery and exercise tolerance.

The patient was scheduled for a follow‐up CPET with the CRT‐D on DDD mode. Heart rate response was reduced, but it progressively and continuously increased with workload (Image B in Figure 2). *V*O_2peak_ was measured at 15.0 mL/min/kg (47% of age‐predicted) [19] equivalent to 4.3 Metabolic Equivalent of Task (MET) [28].

The patient was stratified as high‐risk for cardiac events during exercise according to the American Association of Cardiovascular Prevention and Rehabilitation [29]. The exercise intervention was programmed based on the CPET results and available guidelines [30, 31]. Exercise targets for heart rate and rate of perceived exertion (RPE) were set based on the ventilatory threshold and the respiratory compensation point (Table 1).

**TABLE 1:** Brief summary of CPET results pre–post intervention.

	Variable	Before‐CR	After‐CR	Net change	Delta change (%)
Ventilatory threshold	VT *V*O_2_ [mL/kg/min]	10.9	12	1.10	10.1
	VT workload [W] [watts]	35	44	9.00	25.7
	VT Power‐to‐weight ratio [watts/kg]	0.50	0.62	0.12	24
	VT Mechanical efficiency (W/EE)	17.3%	19.2%	0.02	10.9
	VT Mechanical economy (*V*O_2_/W)	24.89	22.07	−2.82	−11.3
Respiratory compensation point	RCP *V*O_2_ [mL/kg/min]	14.2	16	1.80	12.7
	RCP workload [W] [watts]	61	118	57.00	93.4
	RCP power‐to‐weight ratio [watts/kg]	0.87	1.66	0.79	90
	RCP Mechanical efficiency (W/EE)	20%	33%	0.12	60.7
	RCP Mechanical economy (*V*O_2_/W)	18.67	11.00	−7.67	−41.1
*V*O_2peak_	Peak exercise time [s]	457.00	638.00	181.00	39.6
	*V*O_2peak_ [mL/kg/min]	16.6	17.6	1.00	6.0
	Peak %*V*O_2_ [%*V*O_2max_ predicted]	52%	56%	0.04	7.7
	Peak workload (W) [watts]	78	126	48.00	61.5
	Peak power‐to‐weight ratio [watts/kg]	1.11	1.77	0.66	59
	RER	1.08	1.10	0.02	1.9
	Peak mechanical efficiency (W/EE)	21%	31%	0.10	47.3
	Peak mechanical economy (*V*O_2_/W)	17.03	11.33	−5.70	−33.5

Abbreviations: RCP, respiratory compensation point; RER, respiratory exchange ratio of *V*CO_2_/*V*O_2_; *V*O_2_, oxygen consumption; VT, ventilatory threshold; W, workload.

**FIGURE 3:**
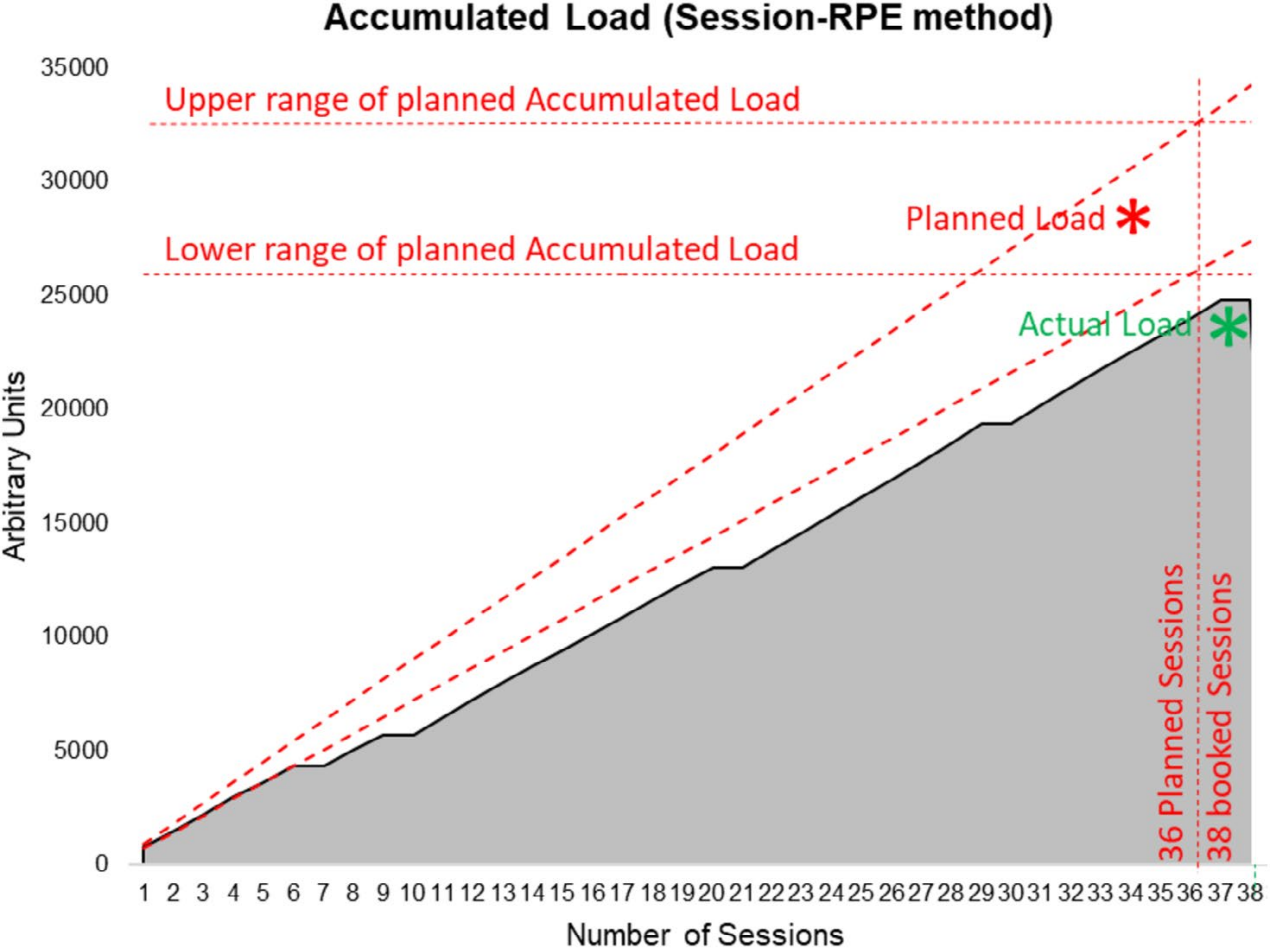
Accumulated load of supervised exercise training across cardiac rehabilitation based on the session‐RPE method.

Progressive overload is a staple of the training process [26, 32]. We evaluated all patients' subjective and objective responses to each workload after each rehabilitation day to confirm they were within prescribed ranges [33]. If needed, the clinical team revised the exercise target heart rate and RPE, and external workloads were adjusted to bring both within prescribed range in the next session. The patient attended 33 outpatient hospital‐based CR exercise sessions with 87% completed as planned. Exercise target heart rate and Session‐RPE targets were met on 94% of sessions. All sessions lasted for the planned duration. Average exercise frequency was 2.6 sessions/week. Exercise training volume quantification was based on the Session‐RPE method [33, 34, 35]. Median daily exercise volume was 720 arbitrary units, and total accumulated training volume was 23,280 arbitrary units (Figure 3).

## 4 Conclussion and Results

**FIGURE 4:**
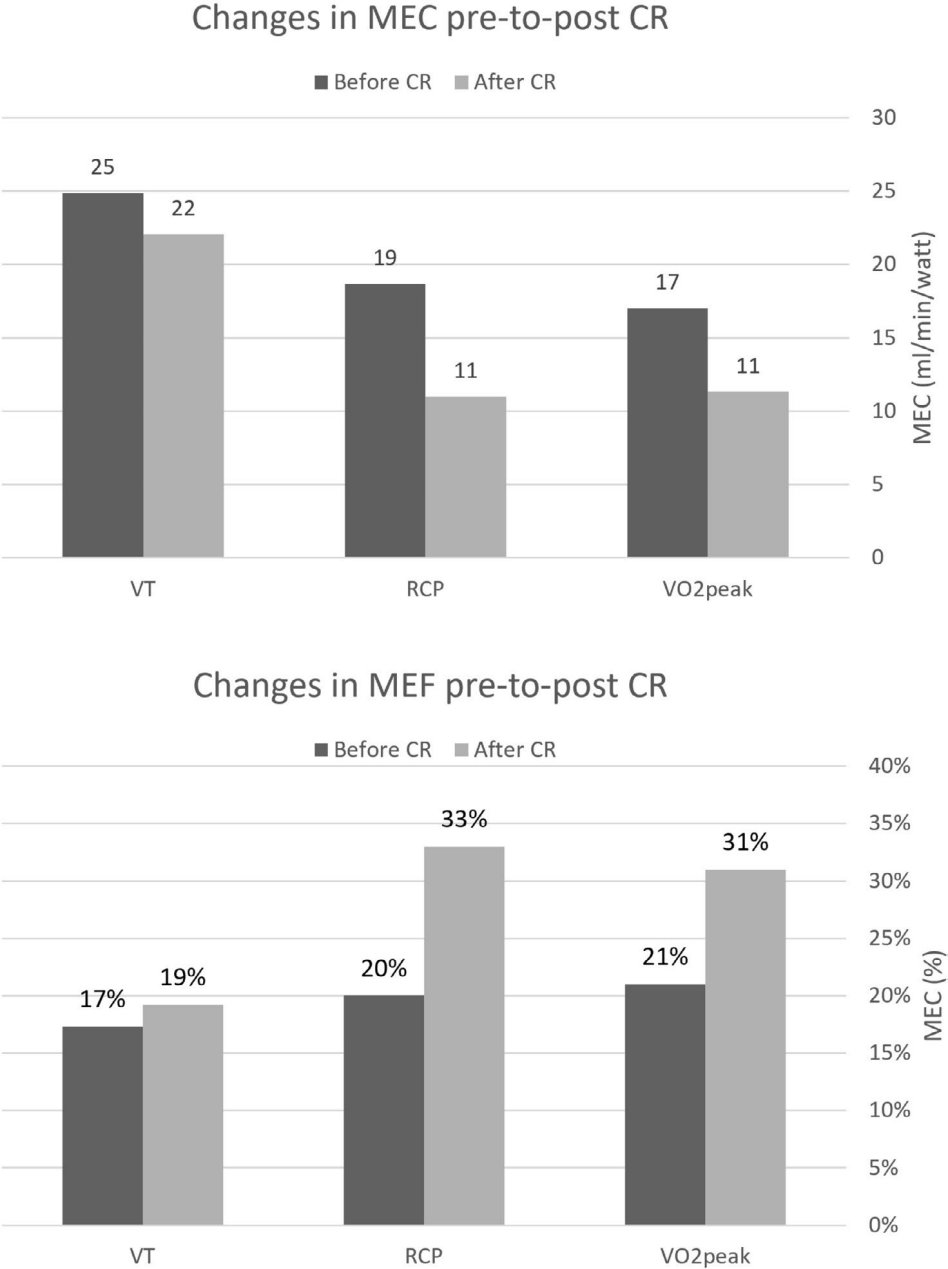
Mechanical economy and mechanical efficiency changes during cardiopulmonary exercise testing ventilatory thresholds before and after cardiac rehabilitation. CR, cardiac rehabilitation; MEC, mechanical economy; MEF, mechanical efficiency; RCP, respiratory compensation point; VO_2_, oxygen consumption; VT, ventilatory threshold.

Pre‐to‐post CR, there was a +9% change in *V*O_2peak_ (1 mL/kg/min, 0.3 MET) and a 61% change in *W*_peak_ (48 W) (Table 1). These can be explained by the improvements in MEC and MEF as underlying physiological determinants of *V*O_2peak_ (Figures 1 and 4). These results highlight the role of peripheral adaptations as a significant driver of exercise performance in patients with HFrEF and CRT‐D, which is clinically relevant and consistent with the evidence. By understanding the concepts of MEC and MEF, clinicians can gain a more accurate assessment of an individual's ability to convert metabolic energy into mechanical work, offering a plausible hypothesis for the commonly seen discrepancy between *V*O_2peak_ and *W*_peak_ changes. Incorporating MEC and MEF into clinical assessments can guide the development of more targeted interventions in patients with HFrEF with a CRT‐D, and therefore MEC and MEF should be routinely measured and reported for assessing rehabilitation outcomes.

## 5 Discussion

*V*O_2peak_ reflects the maximal capacity of the cardiopulmonary system to transport and utilize oxygen during exercise [36]. Absolute *V*O_2peak_ was minimally improved by 0.3 MET (+9%). In HFrEF, *V*O_2peak_ is a strong predictor of mortality, and there is a mortality reduction of 19% with every +1 MET [37]. Every 6% increase in *V*O_2peak_, adjusted for other significant predictors, has also been associated in heart failure with a 5% lower risk of time to all‐cause mortality or all‐cause hospitalization; a 4% lower risk of cardiovascular mortality or cardiovascular hospitalization; and an 8% lower risk of heart failure‐related mortality or hospitalization.

*W*_peak_ was disproportionately increased (+61%) pre–post CR when compared to *V*O_2peak_ (+9%) (Table 1). This apparently disproportionate increase is not uncommon after CR. Although there is a correlation between *V*O_2peak_ and *W*_peak_, the relationship between both can vary depending on the individual and the exercise test protocol. Overall effect sizes for all physiological determinants of exercise performance have not been determined. It is therefore not possible to attribute a conversion rate between a percentage or unit increment in *V*O_2peak_ and an increase in *W*_peak_.

Patients diagnosed with HFrEF typically have reduced systolic volume and a reduced ability to increase their heart rate [38]. This limits their ability to increase cardiac output during exercise. Oxygen pulse (oxygen consumption divided by heart rate) is a limited surrogate measure of the systolic volume and a sensitive marker of abnormalities in the cardiovascular system [16, 39]. Systolic volume can improve with exercise by improving cardiac filling and reducing afterload through improvements in peripheral vascular resistance. Improved vasodilation is critical in patients with HFrEF, as it allows for greater blood flow to the working muscles and improved oxygen delivery. Exercise training can induce this adaptation by improving endothelial function and reducing vascular resistance. It may also improve cardiac contractility, allowing greater force generation by improving calcium handling and increasing the sensitivity of the contractile proteins to calcium [40]. In this case report, oxygen pulse increased by 6%, heart rate peak increased by 11%, and chronotropic index increased by 43% to reach normal range [41].

Peripheral dysfunction can be caused by decreased mitochondrial density, altered fiber type, impaired oxygen delivery, increased oxidative stress, and overall, a decreased contractile efficiency and increased oxygen cost of force generation. Exercise training can improve peripheral function by inducing favorable adaptations in all these factors, leading to improved oxygen extraction and utilization during exercise. This results in increased exercise capacity, more efficient use of energy resources, and decreased fatigue. These adaptations can be indirectly assessed through MEC and MEF during CPET [42].

MEC and MEF during cycling are more uniform between individuals when compared to treadmill exercise [19, 30, 31]. MEF during treadmill is known to vary more significantly due to differences in gait patterns, body weight, and metabolic condition. Despite this variability, treadmill testing was selected over cycling in this case report to accommodate the patient's CRT‐D pacing mode, which is more reliably assessed during upright exercise.

### 5.1 Measuring Mechanical Efficiency During Treadmill Exercise

MEF can be measured as the ratio of W to EE, expressed as a percentage. EE was computed using the formula [43] *E* = (4.94 × RER + 16.04) × (*V*O_2net_, in mL/min) × 60–1. Net oxygen uptake was determined by subtracting resting *V*O_2_ from total *V*O_2_. MEF was calculated in net terms using the following formula [44]: MEF = (W/EE) × 100 − 1.

To calculate W in watts on a treadmill exercise, it is necessary to account for the force exerted by the person, dependent on the velocity at which they are moving, as well as how much of the force is being used to overcome the elevation (expressed either as a percentage slope or converted to an angle) and the gravitational constant (9.81 m/s^2^). This force is primarily determined by the person's weight acting against gravity due to the treadmill's incline, calculated using *F* = m·g·sin(*θ*), where *m* is the patient's mass, *g* is the gravitational constant, and sin(*θ*) is the incline angle of the treadmill. Additionally, to the power required to overcome the incline, the horizontal component of treadmill walking should also account for some energy expended to maintain forward motion. This could be factored into the equation by adding a small rolling resistance coefficient, which, for activities like walking on a treadmill, is considered negligible. These equations theoretically provide a broad explanation of how metabolic energy is efficiently converted into mechanical work within the human biomechanical system.

### 5.2 Measuring Mechanical Economy During Treadmill Exercise

MEC can be derived during a CPET as the ratio *V*O_2_/W. It provides a simple surrogate way to quantify how an individual is using oxygen to produce power. A lower *V*O_2_/W ratio may indicate that the individual can produce more W for a given amount of oxygen consumed, suggesting better MEC. Nonetheless, there are some limitations for the use of *V*O_2_/W, as it is an indirect measure best used for analyzing constant workload steady‐state exercise. *V*O_2_/W infers MEC based on metabolic and mechanical parameters, but gait, muscle fiber type, and training status can affect the relationship between *V*O_2_ and W.

In the present report, MEC is expressed as the gross point estimate of oxygen cost (*V*O_2_/W). Because gross *V*O_2_/W includes the resting or unloaded oxygen uptake, gross MEC may overestimate oxygen cost at low workloads. Alternative approaches in MEC calculations in cycling literature include net MEC, defined as (*V*O_2_ − *V*O_2__0‐W)/W, and the Δ*V*O_2_/ΔWR slope. This slope is calculated across the sub‐threshold linear portion of the test, describing the gain of *V*O_2_ with respect to work rate, and is less sensitive to early and late stage nonlinearity [19]. These alternatives may reduce intercept bias. When interpreting the gross *V*O_2_/W ratios, it should be noted that net MEC or Δ*V*O_2_/ΔW may also yield lower absolute MEC values, although the directional inference in this case would be unchanged. Treadmill test MEC and MEF exhibit greater inter‐individual variability than cycling due to gait and body mass, and results can depend on speed, grade, and stage structure [19, 20]. Indices in this report are best interpreted as within‐subject change under an identical protocol.

### 5.3 Measuring Mechanical Efficiency and Economy at Ventilatory Thresholds

We assessed MEC and MEF at the first ventilatory threshold, the respiratory compensation point, and *V*O_2peak_ (Table 1). The percentage change in MEC and MEF pre‐ to post‐intervention was calculated using the formula: for example: [(Efficiency post − Efficiency pre)/Efficiency pre] × 100 and presented as a percentage. MEC and MEF demonstrated improvement at all points (Table 1, Figure 4). The magnitude of change is in line with previously reported data [25, 42, 45]. MEF improved more than MEC, consistent with a dominant peripheral adaptation [17, 24].

While genetic factors undoubtedly play a significant role, exercise training quality and quantity can influence various aspects such as shifting muscle fiber type, better substrate utilization due to increased cardiorespiratory efficiency (related to *V*O_2max_, heart rate, and minute ventilation), biomechanical efficiency (kinematics, kinetics, flexibility), and neuromuscular efficiency (including neural signaling, motor programming, force production, and muscle tendon stiffness) [20]. In addition, other factors such as muscle mass, fiber type composition, and neuromuscular adaptations could contribute to MEC and MEF improvements. Previous studies in HFrEF have documented increases in muscle mass and shifts towards more oxidative muscle fibers following exercise training [25]. Sympathetic nerve activity targeting skeletal muscle vasculature is also lower after exercise training; therefore, increasing muscle blood flow during exercise. These changes enhance oxygen extraction and utilization at the muscular level, thereby improving exercise capacity. Future research should incorporate direct measurements of muscle morphology and function to better elucidate the mechanisms underlying MEC and MEF improvements [46, 47].

Medications play a crucial role in managing HFrEF and can significantly influence exercise capacity and performance [48]. There were no changes in the patient's medication, including beta‐blockers, which are known to affect heart rate and exercise tolerance. However, the potential impact of medications on the observed outcomes should be considered when interpreting the results.

Considering this single case report, it is not possible to generalize its findings, establish a cause‐effect relationship, nor to determine the magnitude of the effects. Future research should aim to address these limitations by including larger, more diverse samples to provide a more robust understanding of the effects of exercise on MEC and MEF.

Although the *V*O_2_‐W relationship during cycle ramp tests is well described, reference ranges for treadmill‐based gross and net MEC and MEF at defined physiological anchors are sparse and method dependent. Differences in modality, protocol, and analysis windows limit cross‐study comparisons. Future work would benefit from standardized treadmill reporting of gross and net MEC and Δ*V*O_2_/ΔWR, alongside MEF. Studies comparing treadmill versus cycling could further clarify the role of modality‐specific adaptations. Extended follow‐up periods would allow for investigating the long‐term sustainability of the adaptations. Randomized controlled trials with larger samples would be valuable in establishing a clearer cause‐effect relationship between MEC, MEF, and functional capacity in HFrEF. Future work should incorporate echocardiographic and device interrogation data to strengthen the mechanistic interpretation of results.

The findings of this report have high applicability for the clinician when assessing functional capacity changes in HFrEF. This report makes a significant contribution by assessing MEC and MEF as clinically relevant parameters in clinical exercise physiology. This case serves as a methodological foundation for a prospective series to evaluate MEC and MEF in a broader cohort of patients with HFrEF and CRT‐D undergoing CR.

## Author Contributions

**Javier Loureiro Diaz:** conceptualization, data curation, investigation, methodology, project administration, writing – original draft, writing – review and editing. **Praveen Jayaprabha Surendran:** conceptualization, investigation, writing – original draft, writing – review and editing. **Prasobh Jacob:** conceptualization, investigation, writing – original draft, writing – review and editing. **Salma Chbib:** investigation, writing – original draft, writing – review and editing. **Amine Ghram:** writing – review and editing. **Liam David Foster:** investigation. **Omar Ibrahim:** investigation, writing – review and editing.

## Ethics Statement

Patient consent was obtained, and the case report was approved by Hamad Medical Corporation Medical Research Council (MRC) protocol ID MRC‐04‐23‐344.

## Consent

Written informed consent was obtained from the patient for publication of this case report and any accompanying images. A copy of the written consent is available for review by the Editor‐in‐Chief of this journal.

## Conflicts of Interest

The authors declare no conflicts of interest.

## Data Availability

The data that support the findings of this study are available from the corresponding author upon reasonable request.
